# The proto-cortical amygdala: a perspective on the functional and evolutionary relationship between the basolateral amygdala complex and neocortex in mammals

**DOI:** 10.1007/s00429-026-03194-6

**Published:** 2026-09-30

**Authors:** Fionnghuala L. J. James, Sayed K. Ahamed, Cheng Yang, Richard Wilson, Juan J. Canales, Vanni Caruso, Luke R. Johnson

**Affiliations:** 1 School of Pharmacy and Pharmacology, College of Health and Medicine, University of Tasmania, Sandy Bay, Australia; 2 Central Science Laboratory, University of Tasmania, Sandy Bay, Australia; 3 School of Psychological Sciences, College of Health and Medicine, University of Tasmania, Sandy Bay, Australia; 4 School of Health and Environmental Sciences, Auckland University of Technology, Auckland, New Zealand; 5 Center for the Study of Traumatic Stress, Department of Psychiatry, School of Medicine, Uniformed Services University, Bethesda, MD USA

**Keywords:** Basolateral amygdala, Prefrontal cortex, Neocortex, Evolution, Mammals

## Abstract

The basolateral amygdala complex (BNC) plays an important role in learning, memory, motivation, and social behaviour, with its dysfunction implicated in a wide variety of psychiatric disorders. Despite being one of the most intensely researched areas of the brain, much is still unknown about its microanatomical structure and function, relationships and evolutionary origins. In this review, we offer a multidisciplinary approach through the lens of comparative anatomy and evolutionary developmental studies to provide a better understanding of the anatomical and functional relationship between the amygdala and cortex in memory, learning, and social behaviour. Particular attention is paid to the evolutionary origin of pallial areas across amniotes, as well as homology between principal neurons, cortical layers, BNC nuclei, and connectivity between these areas. We argue that the basolateral amygdala complex functions as an early form of the mammalian neocortex. This theory will help to further the understanding of amygdala and cortex function in both health and psychiatric disease.

## Introduction

The amygdala complex, located within the limbic system, is a highly conserved yet structurally heterogenous collection of nuclei key to the linking of sensory information and emotional learning (Janak and Tye 2015; Johnson and Le Doux 2010; Johnson and Doux 2018; Johnson and LeDoux 2004; LeDoux 2007; Martinez-Garcia et al. 2009; Olucha-Bordonau et al. 2015; Pabba 2013; Pitkanen et al. 1997; Sah et al. 2003; Šimić et al. 2021; Swanson and Petrovich 1998). Based on shared anatomical and histochemical features, the mammalian amygdala complex can be roughly split into two main groupings: the corticomedial or ‘superficial’ nuclei, consisting of the central, medial, and cortical nuclei, as well as the nucleus of the lateral olfactory tract (NLOT), and the basolateral or ‘deep’ group, made up of the lateral, basal, and basomedial nuclei (Janak and Tye 2015; Martinez-Garcia et al. 2002; Olucha-Bordonau et al. 2015; Pitkanen et al. 1997; Sah et al. 2003; Šimić et al. 2021; Swanson and Petrovich 1998). The latter group is especially noted for its significant similarities to the deeper layers of the neocortex in multiple aspects, including anatomy, histochemistry, development, and gene expression, as well as in key functions in multiple dimensions of behaviour, leading credence to the possibility that it functions as an early form of neocortex (Butler et al. 2011; Groenewegen et al. 1990; Janak and Tye 2015; Karten 1969; McDonald 2020; Medina et al. 2023; Šimić et al. 2021; Swanson and Petrovich 1998).

There is a close functional relationship between the neocortex and basolateral group (referred to from here on as the basolateral nuclei complex of the amygdala (BNC), especially within the realm of emotional learning. The BNC and neocortex share a number of morphological similarities in terms of neuron structure, distribution, and connection, with a corresponding similarity in terms of functional importance in social behaviour and learning, including emotional processing of aversive states such as stress and anxiety (Groenewegen et al. 1990; Janak and Tye 2015; Johnson and Le Doux 2010; Johnson and Doux 2018; Johnson and LeDoux 2004; McDonald 2020; Pitkanen et al. 2000; Swanson and Petrovich 1998). Within the neocortex, the prefrontal cortex (PFC) has been noted as being especially closely connected with the BNC, being heavily reciprocally connected and forming two of the three key structures involved in the aversive learning circuit, alongside the hippocampal complex (Groenewegen et al. 1990; Janak and Tye 2015; Kim et al. 2011; LeDoux 2007; McDonald 1998; Pitkanen et al. 2000; Sah et al. 2003), to the point that some have suggested it be better studied as a single individual circuit, instead of multiple, separate areas (Kim et al. 2011). The BNC forms multiple reciprocal connections with the neocortex, particularly the midline and orbito-prefrontal cortices, and multiple sensory association areas, as well as the hippocampus, implicating its key function in multiple aspects of behaviour, including thirst, hunger, motivation and emotional learning, social cognition, and reproductive behaviour (Janak and Tye 2015; McDonald 1998; McDonald and Mott 2017; Rowniak et al. 2022; Sah et al. 2003; Šimić et al. 2021) (For further information on the relationship between the PFC, BNC, and hippocampal complex, please refer to the following reviews: (McEwen et al. 2016; Ressler et al. 2022; Shin et al. 2006; Yavas et al. 2019). Therefore, the information transmitted, and its neuronal processing is greatly affected by the activity and reciprocal connections of both cortex and BNC (Groenewegen et al. 1990; Janak and Tye 2015; Kim et al. 2011; McDonald 1998; Sah et al. 2003).

Due to their primary roles in emotional processing, multiple theories on the development of emotion link these two brain regions. For example, the emotional theory of somatic markers centres the amygdala as the key region for emotional processing in the central nervous system (CNS), triggering somatic states from primary emotions due to its earlier maturation before the neocortex (Damasio 1996; Šimić et al. 2021). These somatic states lead to instinctive and rapid responses to stimuli, which are then further elaborated on by the ventromedial prefrontal cortex (vmPFC), generating secondary emotions and more complex cognitive processes from these initial primary states (Damasio 1996; Janak and Tye 2015; LeDoux 2007; Medina et al. 2023; Šimić et al. 2021). Therefore, the amygdala gives rise to the initial instinctive reaction, whereas the cortex exerts top-down control and enables adaptation of this initial reaction (Damasio 1996; LeDoux 2007; Medina et al. 2023; Šimić et al. 2021).

This close functional relationship between the amygdala and cortex is especially important in mediating reward and avoidance behaviours, as well as multiple aspects of social cognition (Emery 2000; Janak and Tye 2015; Kim et al. 2011; LeDoux 2007; Rowniak et al. 2022; Šimić et al. 2021). For example, LeDoux’s higher order theory of consciousness and threat conditioning posits that instinctual behaviours and physiological responses are driven by first-order subcortical structures, such as the amygdala, which then give rise to higher-order and more subjective emotional experiences, which are driven by activity of cortical circuits located in the PFC (LeDoux 2007; Šimić et al. 2021). Therefore, the BNC acts similarly to an early form of neocortex, allowing for the generation of initial reactions before their further cognitive development in the neocortex.

Based on these qualities, there is potential for the BNC and its analogues to perform a similar function as a sort of ‘proto-cortex’ in animals that lack a homologous structure (Butler et al. 2011; Karten 1969; Pabba 2013; Swanson and Petrovich 1998). This review aims to examine similarities between the BNC and neocortex through reviewing current knowledge on the shared evolution and development of the BNC and neocortex and comparing individual nuclei to the six layers of the neocortex in terms of anatomy, structure, histochemical features, and gene expression. Understanding the close relationship between these two areas is crucial for bettering our understanding of their role in psychopathology, such as anxiety and trauma, as disruption of the relationship between these two areas has been identified as relevant in the development of associated disorders, such as anxiety disorders and post-traumatic stress disorder (PTSD) (Johnson and Le Doux 2010; Johnson and Doux 2018; Johnson and LeDoux 2004). Therefore, increasing our understanding of the relationship between these areas may improve our understanding of how these disorders develop, and to identify better and more effective targets for treatment.

### The amygdala

The integration of sensory and emotional information, and the ability to learn from it, is crucial for adaptive functioning in animals. This is exemplified primarily by the BNC’s role in threat learning and avoidance, which plays a crucial role in enabling survival in animals (Janak and Tye 2015; Johnson and Le Doux 2010; Johnson and Doux 2018; Johnson and LeDoux 2004; McDonald 2020; Mesulam 1998; Pine et al. 2021; Šimić et al. 2021).

**Fig. 1:**
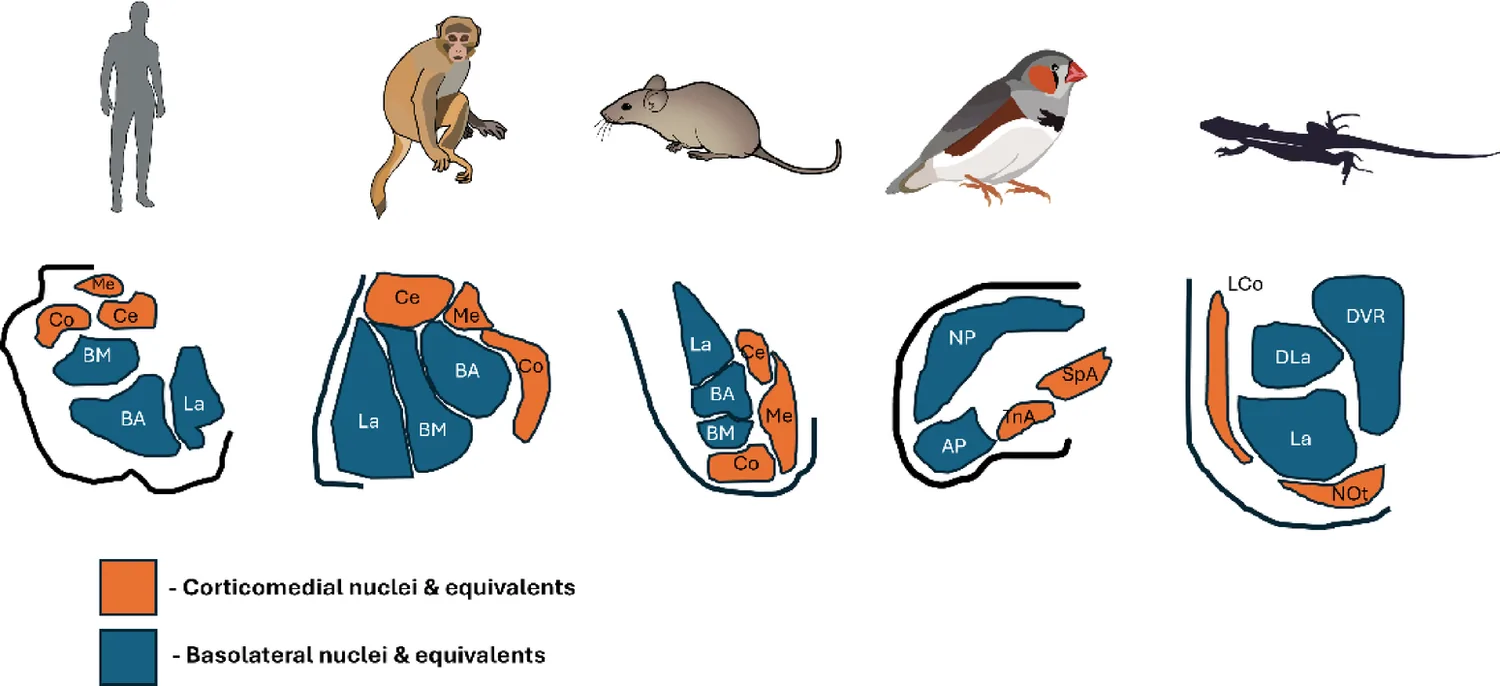
Comparative Anatomy of the BNC complex and its equivalents across amniote classes (coronal view). Comparative anatomy of the BNC complex and its equivalents across amniote classes (coronal view) Me – Medial nucleus of the amygdala, Co – Cortical nucleus of the amygdala, Ce – Central nucleus of the amygdala, BM – Basomedial nucleus of the amygdala, BA – Basal nucleus of the amygdala, La – Lateral nucleus of the amygdala, NP – Nidopallium, AP – Arcopallium, TnA – Nucleus taeniae of the amygdala, SpA – Subpallial amygdala, DVR – Dorsal ventricular ridge, DLa – Anterior subdivision of the dorsal ventricular ridge, LCo – Lateral cortex, NOt – Nucleus of the olfactory tract. Human amygdala referenced from (Grignon 2016), monkey amygdala referenced from (Zikopoulos et al. 2016), mouse amygdala referenced from (Paxinos and Franklin 2001), bird amygdala referenced from (Cheng et al. 1999), and lizard amygdala referenced from (Naumann et al. 2015).

Threat conditioning relies on the ability to rapidly sense, assess, decide, learn and remember an optimal course of action, driven by highly interconnected circuits in the BNC and neocortex (Levy and Schiller 2021). Threat-related behaviours, such as avoidance strategies, are highly conserved across animal classes, showcasing their evolutionary and adaptive importance (Kenwood et al. 2022; LeDoux 2007; Rowniak et al. 2022). The responsible brain structures in the amygdala are also highly conserved, with similar subdivisions in the mammalian amygdala, such as the corticomedial and basolateral groups, also being present in amniotes (vertebrates whose embryos develop in amniotic eggs, such as reptiles, birds, and mammals) and amphibians (Barton et al. 2003; Janak and Tye 2015; Laberge et al. 2006; Medina et al. 2023; Šimić et al. 2021) (Fig. 1).

In mammals, the neural basis of predator response can be traced through shifting activity from the PFC to the midbrain (Levy and Schiller 2021). Two parallel paths encoding defensive behaviours and avoidance originate in the PFC and travel through the cingulate cortex, hippocampus, amygdala, and hypothalamus, before terminating on the midbrain periaqueductal grey (PAG) and dorsal raphe nucleus (Levy and Schiller 2021). Functional anatomical studies from a range of mammalian model species, including rodents, non-human primates, and humans, supports this circuit being conserved in mammals, attributing feelings of anxiety, fear, and cognitive regulation to higher-order cortical areas, and freezing and avoidance behaviours to the BNC (Levy and Schiller 2021; McDonald 2020; Šimić et al. 2021). Within the BNC specifically, the primary pathway mediating threat learning projects from the BNC to the central nuclei of the amygdala (CE), before projections from this nucleus to the rest of the brain, including the neocortex (Martinez-Garcia et al. 2002; Šimić et al. 2021). In turn, the BNC and its homologs in other amniotes are heavily innervated by diverse and significant sensory inputs from multiple brain regions (Butler et al. 2011; Martinez-Garcia et al. 2002; McDonald 2020; Šimić et al. 2021), signalling its importance as a centre for both sensory and emotional processing. The size of the BNC also increases phylogenetically, in parallel with the neocortex and its equivalents, due to these connections (McDonald 2020).

#### Evolution of the amygdala

The origin of the amygdala remains controversial. It shares morphological and histochemical similarities with several other distinct structures in the brain, most notably the neocortex, hippocampus and basal ganglia (Janak and Tye 2015; Johnson and Le Doux 2010; Johnson and Doux 2018; Johnson and LeDoux 2004; Martinez-Garcia et al. 2002; Mesulam 1998; Šimić et al. 2021; Swanson and Petrovich 1998), with evolutionary developmental studies also showing the amygdala expresses genes derived from several other telencephalic regions, including the pallium and subpallium (striatum and pallidum) (Hirata et al. 2009; Martinez-Garcia et al. 2002; Medina et al. 2023; Olucha-Bordonau et al. 2015). Despite this, the amygdala shows a high degree of connective, functional, and developmental coherence across amniotes, with the subdivisions of the amygdala being common to all, supporting the notion that it is a distinct brain structure in its own right (Barton et al. 2003; Johnson and Le Doux 2010; Johnson and Doux 2018; Johnson and LeDoux 2004; Laberge et al. 2006; Martinez-Garcia et al. 2002, 2009; Medina et al. 2023; Pabba 2013; Šimić et al. 2021). Johnston’s 1923 classification dividing the amygdala into two main regions, based on anatomical evidence: the older corticomedial nuclei and the younger basolateral nuclei, remains most in use today (Pabba 2013; Šimić et al. 2021; Swanson and Petrovich 1998). Its exact origins in the ancestral brain remain unclear, though it is generally agreed upon that the BNC, like the neocortex, derives from the pallium, leading to the alternative name of pallial amygdala to refer to this complex (Garcia-Calero et al. 2020; Garcia-Calero and Puelles 2021; Martinez-Garcia et al. 2002, 2009; Medina et al. 2023; Pabba 2013; Puelles et al. 2000, 2019; Yu et al. 2023).

#### Evidence from the embryo

Embryological studies reveal that the pallium develops and organises itself in two distinct ways. The neocortex arises from a layered and organised development, resulting in its six distinctive layers and superficial positioning (Martinez-Garcia et al. 2009). For other pallial structures such as the amygdala, this development results in a nuclear organisation and deeper positioning instead (Garcia-Calero et al. 2020; Martinez-Garcia et al. 2002, 2009). Due to its multiple embryonic origins, the amygdala showcases a mosaic-like structure throughout development, with a diverse abundance of differing cell types (Puelles 2014; Rowniak and Bogus-Nowakowska 2020; Rowniak et al. 2022; Yu et al. 2023). Rowniak and Bogus-Nowakowska (2020) theorised that the expansion of the neocortex in mammals, and the corresponding increase in complexity and information relayed to the BNC required these nuclei to similarly expand in volume, neuron number and density (Rowniak and Bogus-Nowakowska 2020). Reciprocal connections between the two areas would have also increased to strengthen processing power (Rowniak and Bogus-Nowakowska 2020). An example of this can be seen in the differences between the BNC of the common shrew (*Sorex araneus*), a member of one of the most basal placental mammal orders in Eulipotyphla, and the pig (*Sus domesticus*), an artiodactyl noted for their high social intelligence and cognitive abilities. An increase in BNC size, volume, and neuron density can be observed in the pig, with it being approximately 38 times bigger than the shrew BNC, with a corresponding factorial increase in neuron density, relative to body size (Rowniak and Bogus-Nowakowska 2020). Supporting this, Yu and colleagues (2023), based on single nucleus RNA-sequencing data, found highly conserved VGLL3-type glutamatergic neurons in the BNC and its equivalents in humans, macaques, mice and chickens. The high conservation of this neuron type lead the authors to speculate that it is critical to behaviours involved in survival and reproduction (Yu et al. 2023). The BNC specifically is believed to arise from cortical ingrowth and cell migration during development (Olucha-Bordonau et al. 2015; Šimić et al. 2021), whereas more recent evidence suggests that the dorsal pallium, which is the specific region that gives rise to the neocortex, also contributes to the cellular composition of the amygdala (Yu et al. 2023), leading to the similarities in morphology between the BNC and neocortex.

More recent models of pallial development, and its relations to the amygdala, place the BNC as originating from the ventral pallium instead, together with the dorsolateral claustrum, posterior endopiriform nucleus, and the dorsal olfactory cortex (Garcia-Calero et al. 2020; Hirata et al. 2009; Puelles 2014; Puelles et al. 2016, 2019), highlighting its origin and function as an olfactory structure in many animals (with the exception of primates). Various types of ventral pallial cells in the mouse, such as Cajal-Retzius neurons and Pierani cells, show migration into the neocortex throughout development, highlighting the exchange and development these two areas exert on each other, as well as potentially explaining the physical similarities between the two regions (Puelles 2014). Garcia-Calero and Puelles’ (2020, 2021), and Puelles et al. (2019) present an updated radial model of the pallial amygdala, which positions it as a more complex histogenetic region with a nuclear structure, divided into five major domains: lateral, basal, anterior, posterior, and retroendopiriform, with the lateral radial unit containing the majority of the BNC (LA, BA, BM) while the basal unit contains the posterior BNC nuclei, primarily the posterior basolateral (BLP) and posterior basomedial (BMP) nuclei. This model situates the pallial amygdala as self-contained and external to the allocortical ring, which includes the cortical pallium, with a radial organisation perhaps being better suited to improving adaptive and functional fitness (Garcia-Calero et al. 2020; Puelles et al. 2019).

#### Development in primates

In humans, the amygdala has undergone multiple evolutionary changes, resulting in the BNC becoming significantly larger than then corticomedial nuclei, with the LA becoming the largest nuclei of the amygdala (despite its late emergence) due to its role as the afferent and sensory hub of the amygdala (Duvarci and Pare 2014; Fanselow and Gale 2003; Fanselow and LeDoux 1999; Fanselow and Wassum 2016; Garcia-Calero et al. 2020; Janak and Tye 2015; Johnson and LeDoux 2004; LeDoux 2007; Lew et al. 2019; Maren 2003, 2005; Pare et al. 2004; Ressler 2010; Ressler and Maren 2019; Šimić et al. 2021; Tye et al. 2011; Yu et al. 2023). In primates specifically, this enlargement is likely driven primarily by the large number of reciprocal connections between the BNC and neocortex, with the largest afferents to the amygdala originating from the associative cortical fields of the ventral visual pathway, which plays a significant role in the recognition of faces (Lew et al. 2019; Šimić et al. 2021), crucial for social behaviour and signalling in many primate species (Adolphs 2009; Emery 2000; Gallagher and Frith 2003; Lew et al. 2019). This late development and resulting enlargement also parallel the development of the neocortex, being one of the final structures to differentiate itself during the late foetal stage and continuing to undergo a long period of postnatal development (Lew et al. 2019; Šimić et al. 2021).

Within the amygdala, the pallial and subpallial derivatives are adjacent, making the delineation of boundaries difficult without histochemical and cytoarchitectural data (Janak and Tye 2015; Martinez-Garcia et al. 2009; Medina et al. 2023). The pallium and its derivatives, including the pallial amygdala, is generally considered an allocortical structure (Martinez-Garcia et al. 2009; Olucha-Bordonau et al. 2015), though more recent studies considering the radial ring model position it as external to the allocortex (Garcia-Calero et al. 2020; Puelles et al. 2019). The allocortex shows a characteristic organisation consisting of superficial layers, followed by nuclei-like structures deeper to these layers (Olucha-Bordonau et al. 2015), an organisation which resembles the arrangement of the neocortex and amygdala in relation to each other. Furthermore, this arrangement can also be seen within the amygdala itself, with the corticomedial nuclei of the amygdala being superficial, while the BNC is located deeper (Olucha-Bordonau et al. 2015).

This shared pallial ancestry between the neocortex and BNC is also the likely reason for the similarities in anatomy, connectivity, histochemistry, and function between the BNC and neocortex (McDonald 2020; Medina et al. 2023; Olucha-Bordonau et al. 2015; Pabba 2013; Pine et al. 2021; Yu et al. 2023), with some authors such as Swanson and Petrovich (1998) arguing that the BNC should be seen as an extension of the cortex. Due to both being derived from the same ancestral structure, there is potential for the BNC and its homologs in other vertebrates to provide a similar function to the neocortex where this structure is absent (Butler et al. 2011). In more basal vertebrates, such as amphibians, these structures do not show distinct differentiation (Laberge et al. 2006; Laberge and Roth 2007; Pabba 2013), providing more evidence for this theory.

#### The nonmammalian BNC

Despite the high degree of conservation, the pallial amygdala and its derivatives specifically show a large number of divergences between animal classes, making identification of exact homologs difficult (Garcia-Calero et al. 2020; Medina et al. 2023; Yu et al. 2023). Amphibians do not appear to possess a true equivalent to the mammalian BNC, though they do possess a pallial limbic associative structure that shows similarities to the CE in mammals (Laberge et al. 2006), while the LA of amphibians displays similarities to the mammalian BA in terms of sensory inputs (Pabba 2013). Due to the absence of frontal and temporal cortices in amphibians, this limbic component of the brain serves a similar associative function to this area in higher vertebrates (Laberge et al. 2006). Furthermore, the amphibian amygdala still displays an arrangement of nuclei similar to mammals (Pabba 2013), showing that the precursor to amygdala divisions were already in place in amphibians. However, while it is derived from pallial structures, this structure differs developmentally from the BNC and its equivalent in amniotes, therefore it cannot be considered a true homolog (Laberge et al. 2006). The anamniote (fish and amphibians) to amniote (reptiles, birds, and mammals) transition, therefore, was important in establishing key distinguishing characteristics of the BNC, including the establishment and expansion of major thalamic projections to the amygdala and cortex, enabling the integration and processing of multiple channels of sensory, cognitive, and emotional information (Laberge et al. 2006).

In sauropsids (reptiles and birds), the dorsal ventricular ridge (DVR) has been described as an equivalent to both the mammalian neocortex, the claustrum, and pallial amygdala (Butler et al. 2011; Karten 1969; Medina et al. 2023; Pabba 2013; Reiner et al. 2004). It is most homologous to the neocortex in terms of sensory thalamic inputs and motor outputs, but shares more similarities to the claustrum in terms of its position in embryonic development (Medina et al. 2023). In terms of overall connections, the DVR shows most similarity to the BA in mammals: both receive sensory inputs from the thalamic nuclei of the intermediate and ventral tiers, extensive pallio-pallial connections involved in associative memory, and share projections to the basal ganglia, subpallial extended amygdala, hypothalamus, and brainstem regions (Medina et al. 2023). These pallio-pallial connections implicate the DVR in the linking of sensory stimuli and emotional processing, including fear conditioning, as well as social cognition and decision-making (Medina et al. 2023), all key functions that are also shared by the BNC and neocortex in mammals. Gene expression studies during embryonic development link it most closely to the pallial amygdala in terms of transcription factors (*Emx1*, *Emx2*, *Pax6*), with these genes playing key roles in the early differentiation of the pallial amygdala as well as the DVR in sauropsids (Medina et al. 2023). More recent molecular studies have also discovered neuron types present in the sauropsidian pallia, including the DVR, that show a large degree of similarity to neurons in the deep (V, VI), granular (IV), and upper (II, III) layers of the neocortex (Briscoe and Ragsdale 2018; Medina et al. 2023). Another study utilising transcriptomics, however, found a large degree of unexpected divergence between the chicken DVR and mouse neocortex and pallial amygdala, with these differences being attributed primarily to the high degree of divergence between the mammal and avian pallium throughout evolution (Belgard et al. 2013; Medina et al. 2023). Further studies utilising cellular transcriptomics to investigate transcription factor gene expression in glutamatergic neurons of the avian DVR showed significant similarities to the mammalian pallial amygdala but not the neocortex (Colquitt et al. 2021; Medina et al. 2023). However, when this same analysis was expanded to activity-level genes, significant similarities between the DVR and neocortex were noted, suggesting that convergent evolution could be responsible for these similarities (Colquitt et al. 2021; Medina et al. 2023). Data from single nucleus RNA sequencing also shows a high degree of divergence between mammals and sauropsids in terms of BNC excitatory neurons, especially in comparison to subpallial regions of the amygdala, which tend to be more conserved (Yu et al. 2023). However, a high degree of BNC excitatory neuron types, such as SATB2, HGF, and VGLL3, were conserved, especially among mammals, with the diversity and number of excitatory neurons being the highest among primates (Yu et al. 2023).

A high degree of divergence of these structures has also been observed in reptiles. In squamate reptiles, the amygdala equivalent consists of two major centres: the LA and the posterior DVR (pDVR) (Martinez-Garcia et al. 2002; Medina et al. 2023), which has undergone significant evolutionary change to become the nucleus sphericus, a structure significantly involved in vomerolfactory function that is not found in either mammals or birds (Martinez-Garcia et al. 2009; Medina et al. 2023). Similar to their mammalian equivalents, both of these centres give rise to significant glutamatergic projections to the centromedial (striatal in reptiles) amygdala and ventromedial hypothalamus (Martinez-Garcia et al. 2002). In particular, the pDVR/nucleus sphericus, LA, and dorsolateral amygdala (DLA) in reptiles appear to be homologous functionally and anatomically to the BNC of mammals, with the nucleus sphericus showing a similar laminar structure to the neocortex, with a similar ratio of glutamatergic projection neurons and GABAergic interneurons (Martinez-Garcia et al. 2002, 2009). Both structures also showcase large projections to the CE, stria terminalis, and ventromedial hypothalamus (Martinez-Garcia et al. 2009). More crucially, the reptilian DVR and mammalian BNC both share key connectivity, most notably non-chemosensory afferents received from the thalamus and telencephalic sensory areas (in reptiles, these terminate in the anterior DVR), sensory information from the hippocampus, and olfactory information from the lateral cortex (Martinez-Garcia et al. 2009). In squamates, the DLA in particular appears homologous specifically to the BA of mammals in terms of projections, with both projecting bilaterally to the dorsal and ventral striatum/nucleus accumbens (NAc), and NLOT, and receives distinct cholinergic and dopaminergic innervations, both of which are characteristic of the mammalian BA (Martinez-Garcia et al. 2002, 2009). However, the reptilian amygdala does not display the expected reciprocal projections to the dorsal pallium found in mammals, indicating that this characteristic may be a result of the significant changes the dorsal pallium underwent in the course of mammalian evolution, making this characteristic uniquely mammalian (Pabba 2013).

In birds, the identification of the exact homolog to the mammalian amygdala remains controversial, complicated by the expanded presence of the DVR across the surface of the brain (Medina et al. 2023; Reiner et al. 2004). Within the DVR, the avian nidopallium shares several functions with both the mammalian PFC and basal nuclei of the BNC, though its homology is more strictly defined as being related to the basal nuclei only (Medina et al. 2023). In terms of function, the arcopallium specifically appears to be closest to the BNC, as it becomes active primarily in response to fear-related behaviours such as avoidance, whereas both the arco- and nidopallium become active following socio-emotional experiences (Medina et al. 2023). The BSTL in birds, primarily involved in stress response, is also highly innervated with input from both of these areas (Medina et al. 2023). The lack of a neocortex in birds does not disallow similar cognitive and social abilities as seen in primates, with these capabilities being especially developed in parrots and corvids (Butler et al. 2011; Emery 2006; Medina et al. 2023). Common ravens (*Corvus corax*), for example, have shown similar capabilities of theory of mind as seen in great apes, changing caching behaviour in anticipation of competitive behaviour from unseen conspecifics (Bugnyar et al. 2016). The caudolateral subdivision of the nidopallium has been noted to be especially similar to both the mammalian PFC and BA of the BNC, being involved in similar functions such as associative learning, working memory, decision making, and reward encoding (Medina et al. 2023). Martinez-Garcia and colleagues also identify the temporoparieto-occipitalis of the cerebral hemispheres (TPO), dorsal arcopallium (AD), and posterior archstriatum (PoA) as potential analogues to the mammalian BA and reptilian DLA, based on their similar dopaminergic innervations in the striatum (Martinez-Garcia et al. 2009). The integration of cortical and BNC-related functions within the same structure in birds leads credence to the potential of the BNC to act as a neocortical analogue in animals that lack this structure (Butler et al. 2011; Karten 1969). For a table summary of the mammalian BNC and its similarities to equivalent structures in other animals, please refer to Table 1.

**Table 1:** A comparison of the BNC and its equivalents in amniotes

Mammal	Bird	Reptile	Similarities	Amphibian
Basolateral nuclear complex (BNC), consisting of the LA, BA, and BM nuclei	Caudolateral nidopallium Temporoparieto-occipitalis of the cerebral hemispheres (TPO) Dorsal arcopallium (AD) Posterior archstriatum (PoA)	Dorsolateral amygdala (DLA) Posterior dorsal ventricular ridge (pDVR)/nucleus sphericus	Integration of sensory input and behavioural reaction Sensory inputs (thalamus, hypothalamus, brainstem, basal ganglia, striatum and their equivalents) Transcription factors (*Emx1*, *Emx2*, *Pax6)*	No true equivalent; however, the amphibian LA shows similarities to the mammalian BA in terms of sensory inputs

### Relation to the neocortex

The neocortex and limbic system, including the amygdala, both share important functions in terms of attention, memory and learning. Mesulam (1998) posited that the limbic system functioned as both a filter to limit indiscriminate learning in the neocortex, while also being able to learn rapidly to enable adaption to changing environmental conditions, especially in relation to food-seeking and avoidance of danger. This notion is supported by the amygdala’s involvement in both sensory and emotional learning, as well as attention and memory, particularly modulating attention in respect to stimuli with high emotional valence, and to form and process memories associated with this emotional valence (Emery 2000; Lanuza et al. 2008; McDonald 2020; Mesulam 1998; Pine et al. 2021; Pitkanen et al. 1997). This ability for the amygdala to act as an early repository for transient forms of memory, learning, and attentional processing before the emergence of the neocortex supports the notion of the amygdala serving a “proto-cortical” function.

The amygdala and neocortex share several important projections (Martinez-Garcia et al. 2009; McDonald 1998). Within mammals, the associated circuitry between the BNC and neocortex remains highly conserved. Early cortical and subcortical structures, such as the amygdala, communicate sensory information in parallel to multiple neocortical areas, allowing for the analysis of different stimuli (Adolphs 2009; Kenwood et al. 2022; McDonald 1998). In turn, important cortical sensory inputs project back to the BNC, with minor but long descending projections to the CE (Martinez-Garcia et al. 2009; McDonald 1998), thereby forming a feedback pathway (Martinez-Garcia et al. 2009). Early retrograde tract tracing experiments in cats found that the entorhinal cortex projects significantly into the amygdala, with neurons in the deep layers of the cortex projecting directly to the CE and BNC (Martinez-Garcia et al. 2009), with the majority of sensory input to the BNC specifically arising from Layer V principal neurons (Sah et al. 2003). These projections were also found to be topographic, with neurons in the medial entorhinal cortex projecting to the medial subnuclei of the BNC and CE (Šimić et al. 2021). In particular, significant sensory cortical areas such as the olfactory, gustatory, and insular cortices, secondary visual, auditory, and somatosensory cortical areas, and the hippocampus and parahippocampal structures show significant projections to the amygdala (Martinez-Garcia et al. 2009; McDonald 1998, 2020; Olucha-Bordonau et al. 2015). This circuitry is crucial to identifying and coordinating responses to threats, with the BNC enabling fear acquisition and the neocortex regulating and extinguishing these fear responses when they are no longer required (Kenwood et al. 2022; Levy and Schiller 2021; Martinez-Garcia et al. 2009). However, the amygdala-cortex relationship is not restricted to fear learning. Projections from the BNC to the ventral striatum also play a role in reward learning, generating reactions of attraction or reward to incoming stimuli (Janak and Tye 2015; Martinez-Garcia et al. 2009; Pine et al. 2021) showcasing the amygdala’s importance to associative learning in general (Levy and Schiller 2021; Martinez-Garcia et al. 2009).

The expansion of these areas in primates is also thought to be related to their increased importance in social behaviour and cognition (Fig. 1) (Emery 2000; Gallagher and Frith 2003; Kenwood et al. 2022; Lew et al. 2019). In primates, the amygdala, striatum and orbitofrontal cortex (OFC) circuit primarily controls and guides complex decision-making behaviour by encoding task-specific information in both aversive and appetitive situations, enabling the selection of different responses (Adolphs 2009; Emery 2000; Ghashghaei and Barbas 2002; Kenwood et al. 2022; Pine et al. 2021). This circuit, together with the insula, is also highly sensitive to contextual information in social situations, giving rise to the capabilities of feeling empathy, modulated by the insula and premotor cortex, and theory of mind, primarily modulated by the mPFC and tempoparietal junction (Adolphs 2009; Emery 2000; Gallagher and Frith 2003; Pine et al. 2021). In addition to this, the amygdala and orbitofrontal cortex also form a second circuit with the superior temporal sulcus (STS) encoding and processing visual attention in primates, another important cue in social behaviour (Emery 2000). The BNC in particular also shows a large number of reciprocal connections to the neocortex in primates, with the largest afferents to the BNC originating from the associative cortical fields of the ventral visual pathway, which primarily processes facial information, including recognition (Ghashghaei and Barbas 2002; Olucha-Bordonau et al. 2015; Šimić et al. 2021). Neuroimaging studies in humans have further supported this, with activation in the amygdala being recorded in response to facial expressions, especially those perceived as fearful or happy (Olucha-Bordonau et al. 2015; Šimić et al. 2021). The amygdala has also been implicated in the potential development and function of theory of mind due to its rapid activation to socially salient stimuli in primates (Emery 2000). However, fMRI studies of theory of mind show lower activation in the amygdala compared to the neocortex, suggesting that this role has been occupied primarily by the neocortex and related areas in primates (Emery 2000). Differing responses in social behaviour can also be localised to specific nuclei in non-primate mammals: increased stimulation of the BNC and medial nuclei of the amygdala has been linked to an increase in aggressive behaviour, whereas decreased stimulation of these same areas lead to an increase in submissive and prosocial behaviour in rodent models (Šimić et al. 2021). The parallel expansion of the neocortex and BNC, particularly the LA, in primates may have also increased the number of excitatory neuron migrations from the neocortex to the BNC in development, resulting in the similarity in morphology and a subsequent integration of sensory input and cognitive function, leading to the increasing sophistication of these abilities in primates (Rowniak et al. 2022; Yu et al. 2023). Therefore, these examples illustrate how important the amygdala is to social and cognitive processing, especially in primates, fulfilling a similar role to the neocortex alone.

Despite the lack of laminar organisation, neurons of the BNC display a strong similarity in terms of morphology, physiology, and pharmacology to those in the neocortex (Johnson and Le Doux 2018; McDonald 2020; Olucha-Bordonau et al. 2015; Swanson and Petrovich 1998). In contrast to the corticomedial nuclei of the amygdala, the BNC displays a very high proportion of pyramidal-shaped glutamatergic projection neurons, with a corresponding low density of GABAergic interneurons, with the majority co-expressing neuropeptides or calcium-binding proteins (McDonald 2020; Olucha-Bordonau et al. 2015). This pattern of neuron morphology and distribution is also displayed in the neocortex (McDonald 2020; Olucha-Bordonau et al. 2015).

### Basolateral amygdala nuclei and their cortical counterparts

The BNC is characterised by its similarities to the neocortex in terms of neuron morphology and histochemistry, as well as its extensive interconnections to higher-order cortical areas (Janak and Tye 2015; Kenwood et al. 2022; Kim et al. 2011; McDonald 2020; Sah et al. 2003). Due to a shared developmental origin, the BNC displays strong similarities to other pallial structures, including the neocortex, as well as the hippocampus, in terms of neuron morphology and heavy reciprocal connections to each of these areas (Martinez-Garcia et al. 2009; Medina et al. 2023). Within the amygdala, the BNC complex also receives the bulk of inputs from the cortex, with reciprocal projections terminating in the PFC, posterior and anterior insular and perirhinal cortices, and the entorhinal cortex (Ghashghaei and Barbas 2002; Martinez-Garcia et al. 2009; McDonald 1998, 2020; Olucha-Bordonau et al. 2015; Pitkanen et al. 2000; Sah et al. 2003).

Projections to layers II, III, and V of the perirhinal cortex are particularly prominent, with these contributing to emotional-sensory processing, as well as being a potential gateway for this information to the hippocampus (Olucha-Bordonau et al. 2015). In primates (specifically, rhesus macaques), the BNC also shows projections to the anterior medial thalamic nucleus, with these specifically arising from the LA, BA, and BM (Olucha-Bordonau et al. 2015; Xiao and Barbas 2002). Axons originating from area O12 of the orbitofrontal cortex also terminate in all BNC nuclei in the rhesus macaque (Ghashghaei and Barbas 2002). These cortical inputs play a key role in threat learning and also act as the main sensory gateway to the rest of the amygdala, particularly the LA nucleus (McDonald 2020; Pitkanen et al. 1997; Šimić et al. 2021). Due to these qualities, the BNC is particularly important in both generalising and discriminating between sensory cues and fear responses (McDonald 2020; Medina et al. 2023; Šimić et al. 2021). Through connections with the hippocampus and striatum, the BNC also modulates reward memory and motivation via dopamine transmission, originating from the mid-brain and heavily innervating the striatum, whereas its links to the neocortex enables it to exert a large amount of influence on cognition, executive function, perception, and attention, especially to emotionally salient stimuli (Adolphs 2009; Gallagher and Frith 2003; Ghashghaei and Barbas 2002; Janak and Tye 2015; Lanuza et al. 2008; Martinez-Garcia et al. 2009; McDonald 2020; Medina et al. 2023; Olucha-Bordonau et al. 2015; Pine et al. 2021). The mPFC is especially important as a connecting site with the BNC, with a majority of amygdala connections to this area being concentrated in the BNC (particularly the LA and BA nuclei), playing a key role in modulating the activity of the BNC, especially in relation to fear and avoidance behaviours, and extinction learning (Gallagher and Frith 2003; Ghashghaei and Barbas 2002; Janak and Tye 2015; Kim et al. 2011; Olucha-Bordonau et al. 2015; Pine et al. 2021). Fear responses generated in the BNC are brief, usually lasting for a few seconds at most (Pine et al. 2021). The mPFC, particularly the dorsal region, is key to maintaining and prolonging threat responses enough to enable learning, with the adjacent infralimbic (IL) PFC mediating this fear response during extinction learning in rodent models (Kim et al. 2011; Olucha-Bordonau et al. 2015; Pine et al. 2021; Sierra-Mercado et al. 2011). Retrieval of extinction memory involves inhibitory circuits in both the BNC and PFC, with an increase in output activity from the PFC to the BNC (Kim et al. 2011; Pine et al. 2021). Several studies have implicated this wider neocortical connection in emotional memory and response, interestingly regardless of whether the event was actually experienced or imagined (Mesulam 1998; Pine et al. 2021). Therefore, BNC-cortical connections have a strong impact on decision making, especially in relation to classical conditioning (Janak and Tye 2015; Kim et al. 2011; Olucha-Bordonau et al. 2015).

The BNC contains the largest neurons in the amygdala, and due to their close resemblance to neocortical neurons in morphology, synaptology, pharmacology, and histochemistry, this complex is often termed the ‘cortex within the amygdala’ (Janak and Tye 2015; McDonald 2020; Šimić et al. 2021; Swanson and Petrovich 1998). This similarity is especially pronounced with the neurons in the deep (V-VI) and granular (IV) layers of the neocortex, with this similarity also being conserved in birds and reptiles (Medina et al. 2023). Principal neurons showcase a pyramidal or semipyramidal somata shape (leading to their alternative name of pyramidal neuron), and dense spiny dendrites (McDonald 2020). The primary function of these neurons is to reach long-distance targets elsewhere in the brain, with their axons innervating the stria terminalis, ventral amygdalofugal pathway, external capsule, and neocortex (McDonald 2020). These neurons are also heavily interconnected with neighbouring principal neurons and their associated interneurons, via axons innervating the dendritic spines and dendrite shafts of their neighbours (McDonald 2020). Principal neurons typically contain a singular thick dendrite, resembling those in the neocortex, surrounded by thinner dendrites, which more closely resemble basal dendrites of certain principal neurons in the neocortex, though the ease of separation between these dendrites varies (McDonald 2020). Therefore, BNC principal neurons showcase anatomy in common with both newer neocortical neurons and older, basal neurons, common with the BNC showcasing morphological features common to both areas. Specific subtypes of projection neuron, such as LAMP5 + and SATB2+, are abundant in both the BNC and neocortex and share the pallial developmental marker *Emx1*, particularly in primates (Yu et al. 2023).

**Fig. 2:**
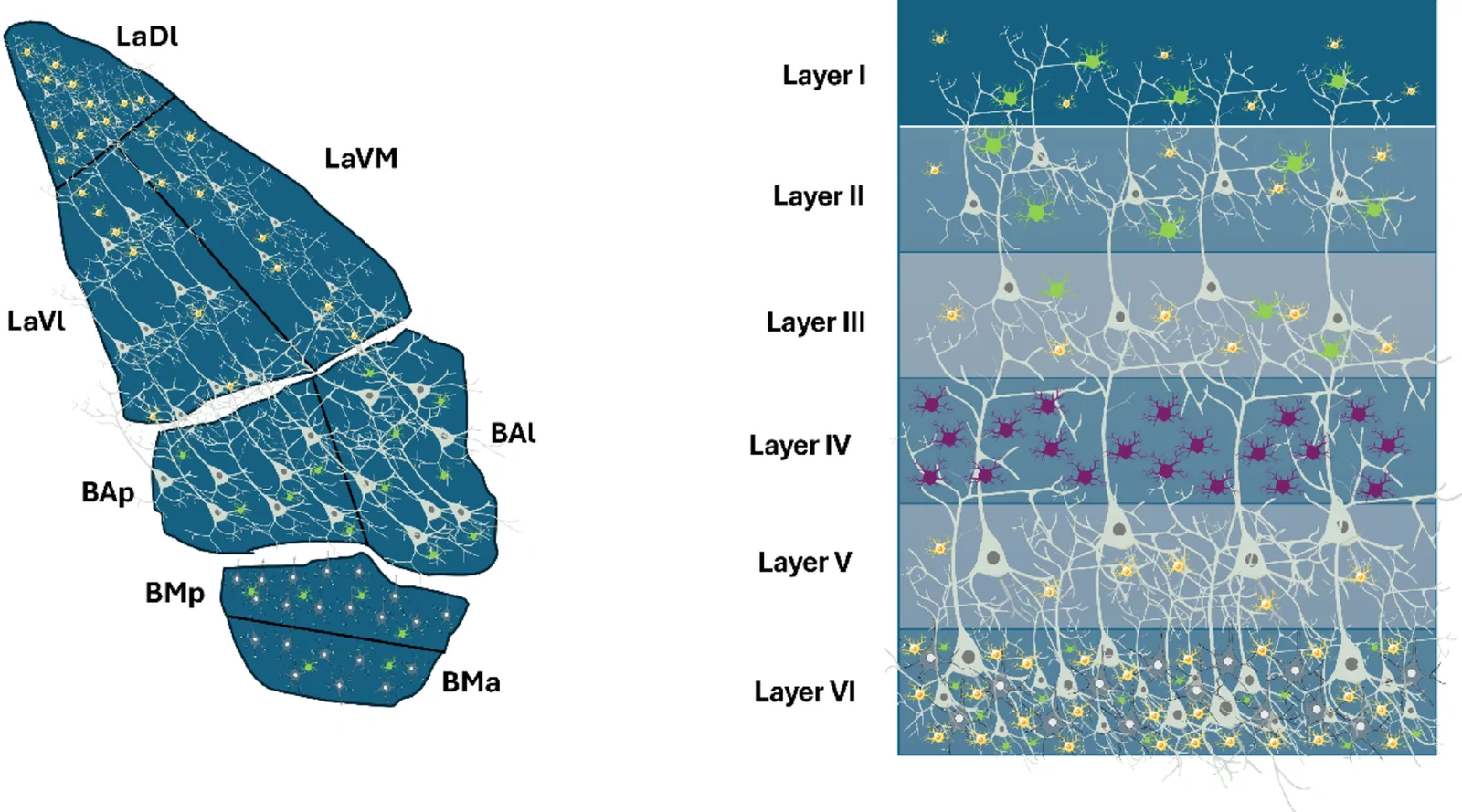
Comparative Neuron Anatomy, Type, and Distribution Between the Rodent BNC and Cortical Layers. Principal neurons are represented as pyramidal in shape with long axons. Interneurons are represented as circular in shape with short dendrites. Different colours mark different categories of neurons. Information for this figure has been taken from Johnson & LeDoux (2018) and McDonald (2020)

The remaining 10–20% of the population are non-principal neurons (NPNs), of which the majority are GABAergic interneurons (INs), which form a partnership with principal neurons and serve as inhibitors, enabling modulation of activity within the neuron pair (Janak and Tye 2015; Johnson and Le Doux 2018; McDonald 2020; Šimić et al. 2021). These neurons differ morphologically from principal neurons in being much smaller, with spherical somata and spine-sparse dendrites, qualities they also share with NPNs in the neocortex (McDonald 2020). The inputs to these INs are excitatory and are mostly local, with few also receiving longer-range inputs from the neocortex and thalamus (Sah et al. 2003). This ratio of neuron type is also similar to that found in the neocortex, with around 70–80% of the neocortex consisting of glutamatergic principal neurons, with the remaining 20–30% consisting of INs (Kenwood et al. 2022). In rats, IN subpopulations in the frontal neocortex closely resemble those in the BNC (McDonald 2020). Within the BNC, the majority of INs use parvalbumin (PV+) as a calcium-binding protein, with their inhibitory interactions with principal neurons largely determining overall neuron activity in this area, especially in terms of gamma and theta rhythms (McDonald 2020). INs in the neocortex also exert the same inhibitory effect on principal neurons, and in doing so, modulate neuron activity (Kenwood et al. 2022). Consistent with the close relationship between the BNC and PFC, PFC neurons, particularly in primates, also play a key role in the linking of sensory input and cognitive processing (Mesulam 1998), a function similar to the BNC. This connected network activity and oscillations in activity between principal neurons and INs are critical in the BNC’s role in synaptic plasticity and the formation of emotional memories (McDonald 2020). A summary and comparison of the broad neuron types and their distribution throughout the BNC and cortical layers can be seen in Fig. 2.

**Table 2:** A Comparison of Key Characteristics Between BNC Nuclei and Suggestions on Their Cortical Equivalents

BNC Nucleus	Developmental genes	Connectivity	Neuron Anatomy	Function	Cortical equivalent
LA	Emx1 Pax6 Tbr1 Lmo3	*Receiving* Auditory thalamus Auditory cortex (Secondary, associative sensory) Visual cortex (Secondary, associative sensory) Somatosensory cortex (Secondary, associative sensory) Insular cortex Subiculum Entorhinal cortex Layer V pyramidal neurons (neocortex) PFC Piriform cortex (posterior) Perirhinal cortex Medial temporal lobe Posterior intralaminar complex Hippocampus Posterior internuclear nucleus (PIN) Gustatory thalamic nucleus Parvicellular region of the posterior ventral thalamic nucleus Visceroceptive thalamic nuclei Hypothalamus Thalamus Nucleus accumbens *Projecting* BM BA Periamygdaloid complex CeDM (largest projection) CoP Capsular CE AHAla Intercalated cells of the amygdala Prefrontal cortex (PFC) Perirhinal cortex (Layers II, III, V) Medial temporal lobe Nucleus accumbens (NaC) Striatum (dorsal)	Types: Glutamatergic principal (SAT52+, LAMP5+) Size: Principal: Small to medium, pyramidal in shape, greatly resemble pyramidal neurons in the cortex. Firing properties: Broad firing (95%), single firing (3%), short duration action potential (2%) Calcium-binding proteins: Calbindin, calretinin, parvalbumin Neuropeptides: Somatostain, enkephalin Staining properties: Less reactive to AChE staining compared to neurons in the BA	Emotional responses Emotional learning Sensory interface of the BNC	Function: Layer IV (primary sensory input area, heavily innervated by the thalamus) Neuron anatomy: Layer V
BA	Emx1 Lhx9 Pax6 Tbr1 Lmo3 Lmo1 Lmo4	*Receiving* LA Auditory cortex (Secondary, associative sensory) Visual cortex (Secondary, associative sensory) Somatosensory cortex (Secondary, associative sensory) Insular cortex Gustatory thalamic nucleus Visceroceptive thalamic nuclei Mediodorsal thalamic nucleus Oc2 (cortical connections terminate in this nucleus) PFC (main target of afferents from this area) Piriform cortex (posterior) Perirhinal cortex Entorhinal cortex Subiculum Medial temporal lobe Hippocampus Nucleus accumbens Basal forebrain (Cholinergic afferents) Dopaminergic cell groups of the ventral tegmental area-substantia nigra *Projecting* CeM (largest projection) Intercalated cells of the amygdala Medial temporal lobe PFC (mPFC, afferents, preferentially connect with GABAergic PV+ neurons in the PFC) Perirhinal cortex (Layers II, III, V) Hippocampus (stratum oriens, stratum radiatum CA3, CA1, ventral) Thalamus (mediodorsal thalamic nucleus) Striatum (ventral, dorsal) Nucleus accumbens	Type: Glutamatergic principal (95%) (LAMP5+) Firing pattern: Burst firing, repetitive firing Size: Larger than LA Type: GABAergic interneuron (5%)	Emotional responses Emotional learning	Layer V shows the broadest similarities to the BA in terms of neuron anatomy, distribution, and function, along with the CE. Layer VI also shares the same projections to the thalamus as seen in the BA.
BM	Emx1 Pax6 Tbr1	*Receiving* LA CeM PFC (afferents) Perirhinal cortex BNST vmPFC Piriform cortex (posterior) Insular cortex Medial temporal lobe Posterior internuclear nucleus (PIN) efferents Visceroceptive thalamic nuclei Nucleus accumbens Extensive rostrocaudal connections *Projecting* CE Perirhinal cortex (Layers II, III, V) Medial temporal lobe Nucleus accumbens Striatum (Anterolateral, ventral, dorsal) Hippocampus (Stratum lacunosum-moleculare of CA1)	Type: Glutamatergic (LAMP5+) Size: Small and densely packed in BMa, large and loose in the BMp Notes: NADPH staining strongest in this nucleus. Less reactive to parvalbumin and GABA Most diverse and divergent neurons out of the BNC	Emotional responses Emotional learning	Neuron morphology and diversity: Layer VI. Layer Vb in the primate visual cortex also shows particular similarity to the BMp in terms of principal neuron anatomy (large and solitary, distinguished by a single apical dendrite and numerous large basal dendrites) Function: Layer V, similar to the BA

#### LA (lateral nucleus of the BNC)

**Fig. 3:**
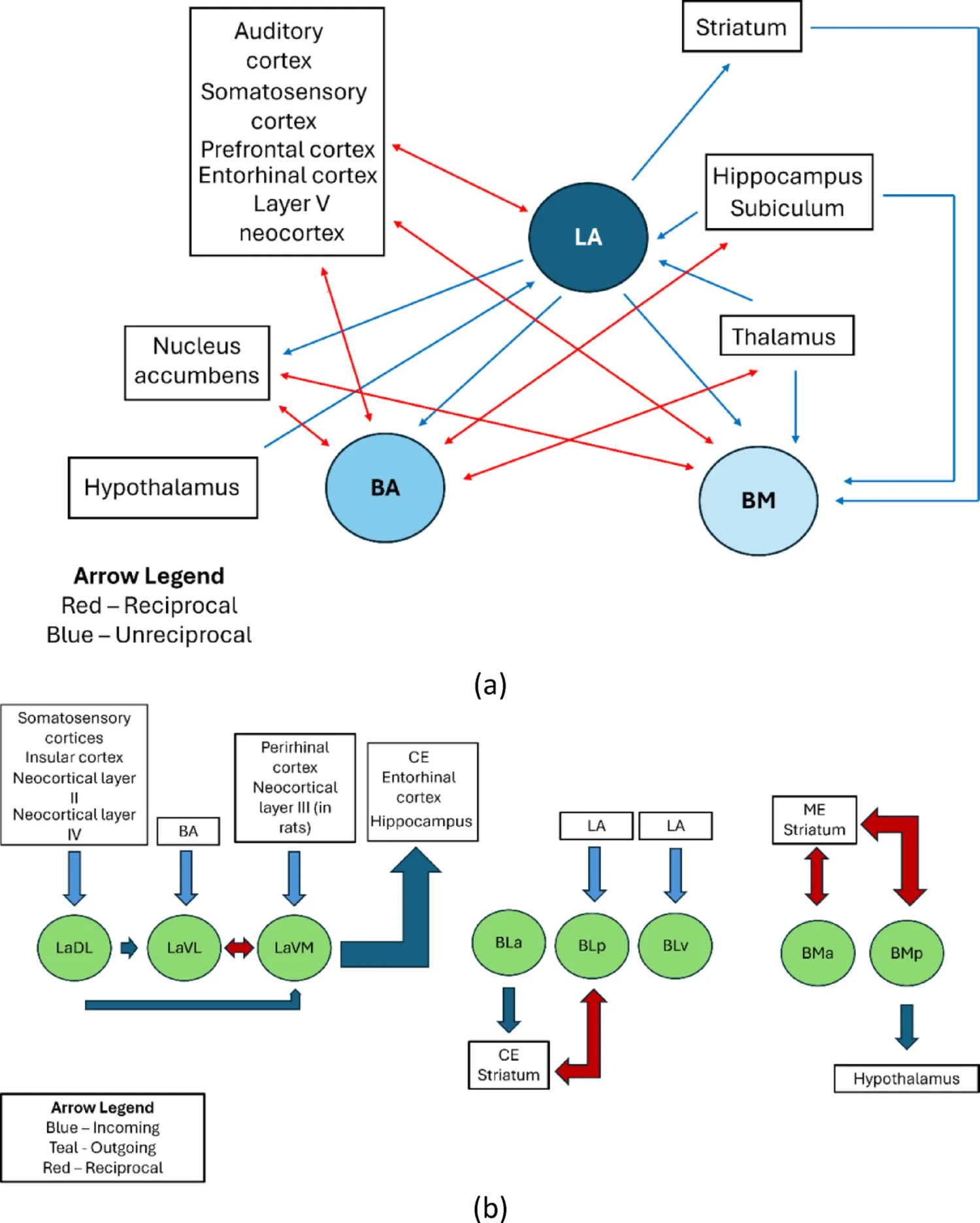
**a** Simplified Connectivity Between BNC Nuclei and Their Main Input and Output Sources. Red arrows denote reciprocal connections, while blue arrows denote unreciprocated connections.** b** BNC Subnuclei-specific Connections Both Within and External to the Amygdaloid Complex. Blue arrows denote incoming connections, whereas teal arrows denote outgoing ones. Red arrows denote reciprocal connections

The LA, consisting of the dorsolateral (LaDL), ventrolateral (LaVL), and ventromedial (LaVM) subnuclei, is the largest nucleus in the human amygdala, extending across its entire length (Olucha-Bordonau et al. 2015; Šimić et al. 2021). It is the main afferent structure and sensory gateway for the amygdala, with the majority of afferent fibres and strongest projections originating from this nucleus (Olucha-Bordonau et al. 2015; Pitkanen et al. 1997; Šimić et al. 2021). It receives strong topographic projections from the neocortex, which project further onwards to its subnuclei, as well as other amygdala nuclei, most notably the CE (Pitkanen et al. 1997; Šimić et al. 2021). A number of these cortical connections project back to the LA, forming a loop of information flow between the BNC and neocortex (McDonald 1998; Šimić et al. 2021) (Fig. 3). It is the key structure implicated in fear learning and memory, particularly the processes of memory consolidation and reconsolidation, where memories are labilised and strengthened into a long-term, persistent form, attracting interest as a target for potential therapeutics in trauma and anxiety disorders (Bergstrom and Johnson 2014; Bergstrom et al. 2011; Šimić et al. 2021).

Its subnuclei are intrinsically connected, and it also possesses strong, reciprocal glutamatergic connections to the other nuclei of the BNC, the corticomedial and periamygdaloid nuclei, as well as strong, but unreciprocated, projections to the CE (Pitkanen et al. 1997; Šimić et al. 2021). The majority of these reciprocal connections terminate in the LaVL and LaVM, though not the LaDL, which is primarily connected to the other two LA subnuclei (Olucha-Bordonau et al. 2015; Pitkanen et al. 1997; Sah et al. 2003). However, the LaDL does receive projections from the ventral visual stream via the anterior and inferior temporal cortices, implicating a role in novel object recognition (Ghashghaei and Barbas 2002; McDonald 2020), as well as strong efferents from primary somatosensory areas, including the primary gustatory and visceral cortices, which are primarily segregated in this subnucleus (McDonald 2020; Sah et al. 2003). Principal neurons in cortical layers II and IV also project heavily to the LaDL, along with subcortical inputs from the thalamic medial geniculate nucleus (Sah et al. 2003). These projections play a key role in conveying sensory information, including from auditory, somatosensory, and visual sources, to the LA and the CE, playing a key role in fear conditioning (Bergstrom and Johnson 2014; Ghashghaei and Barbas 2002; Olucha-Bordonau et al. 2015). Unlike the LaDl and LaVL though, the LaVM is distinguished by its inputs primarily originating from primary cortical regions involved in processing sensory information (particularly the perirhinal cortex), rather than direct connections to sensory areas, leading to a muted response in this subnucleus compared to the other LA subnuclei (Pitkanen et al. 1997; Sah et al. 2003). Polymodal projections and afferent connections that play a function in declarative memory are also limited to this subnucleus (Sah et al. 2003). Therefore, the combination of this features suggests the importance of the LaVM as a hub for integrating sensory information with memories of past experiences (Sah et al. 2003), similar in function to layer IV of the neocortex. In rats, this subnucleus also shares common connectivity with neocortical layer III, particularly in the entorhinal region, with PNs in this subnucleus projecting directly to this layer (Olucha-Bordonau et al. 2015).

In common with the CE, weak projections are also sent to the amygdalohippocampal area (McDonald 1998; Pitkanen et al. 1997; Šimić et al. 2021). The BA also sends distinct inhibitory and excitatory projections to the LaVL (Pitkanen et al. 1997).

The majority of research into LA connectivity in rodents primarily concerns its role in auditory fear conditioning, with the LaDL and LaVL receiving direct inputs from the auditory thalamus (Pitkanen et al. 1997). Pathways that process the conditioned stimulus (CS) and unconditioned stimulus (US) used in fear conditioning paradigms converge onto multiple synapses in the LA, travelling via the medial geniculate body of the thalamus, and Brodmann’s areas 1, 2, 3, 41, & 42 of the neocortex (Bergstrom and Johnson 2014; Šimić et al. 2021). It is also the primary target for sensory input from cortical areas in the amygdala, which terminate in all subnuclei in the LA (Pitkanen et al. 1997). Among these, projections from the auditory cortices primarily terminate in this nucleus and the anterior subnuclei of the BA (McDonald 2020), which are particularly important in the BNC’s role in auditory fear conditioning. Two main afferent pathways connect to the amygdala through the LA: a faster, ‘low-road’ direct pathway bypassing the neocortex, enabling immediate reactions, and a slower, ‘high-road’ pathway travelling through the thalamus and neocortex before reaching the LA, allowing for more considered reactions (Bergstrom and Johnson 2014; Bergstrom et al. 2011; Šimić et al. 2021). Efferent projections outside of the amygdala are sent to the OFC, medial PFC (mPFC), and ventral striatum, with neuron populations in the NaC being the largest target of these connections (Ghashghaei and Barbas 2002; Sah et al. 2003; Šimić et al. 2021).

**Fig. 4:**
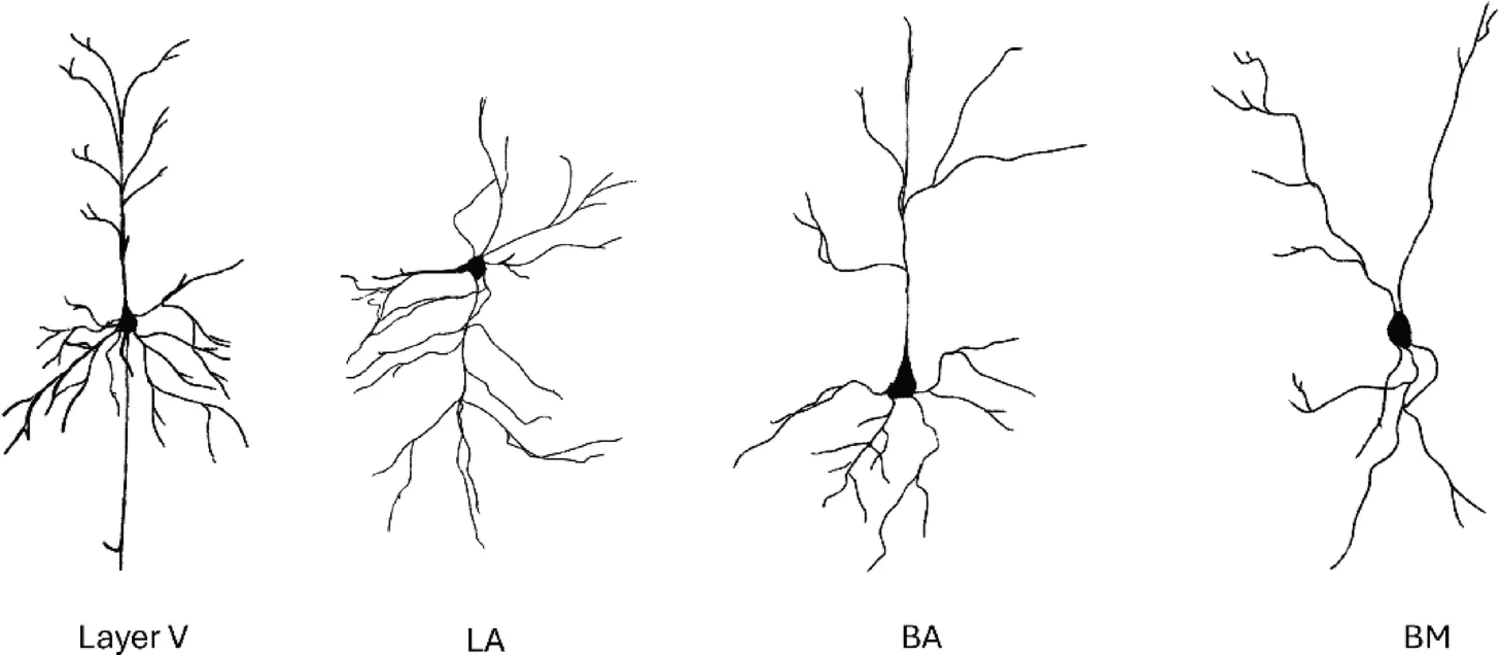
Comparative Anatomy Between Typical Principal Neurons from Neocortical Layer V and BNC Nuclei. Principal neurons in the deep cortical layers and BNC nuclei show remarkable morphological similarities to each other. Neurons in the BA show a particular closeness to cortical neurons. LA neurons are also similar, though are less oriented and tend to be smaller in size. The BM is the most deviant from the cortex, being the smallest of the BNC and showing much sparser branching of the dendrites compared to the cortex and rest of the BNC. These illustrations were created based on the author’s own data and references from McDonald (2020). Neurons are not drawn to scale.

Its neurons are largely similar to those found in the deep neocortical layers, though the principal neurons of the LA are smaller in size (Olucha-Bordonau et al. 2015) (Fig. 4). The LaDL shows the smallest neurons in the LA, while the LaVL shows the largest, with the LaVM being intermediate between these subnuclei (Sah et al. 2003). LA subnuclei can be distinguished by their neurons based on their differential staining, with the LaDL and LaVl showing the most intense acetylcholinesterase staining, whereas the LaVM is more reactive to the Timm’s method (Olucha-Bordonau et al. 2015). Excitatory input in this nucleus is received from glutamatergic neurons, with these inputs stimulating NMDA and AMPA receptors in both principal neurons and INs (McDonald 2020; Šimić et al. 2021). Principal neurons in this nucleus show a variety of firing properties, yet little difference in synaptic properties, suggesting that these inputs to LA principal neurons are largely the same, a similarity shared with the neocortex (Sah et al. 2003). However, unlike the neocortex, these differences are not coupled with drastic morphological differences (Sah et al. 2003). Specific neuron populations in the LA modulate fear (Bergstrom and Johnson 2014; LeDoux 2007; Lee et al. 2017; McDonald 2020), and reward reactions via phasic activation, whereas anxiety is linked to persistent and long-term changes in overall neuron activity (LeDoux 2007; Lee et al. 2017; Šimić et al. 2021). Specific, highly excitatory populations of principal neurons in the LA also encode memory engrams associated with auditory fear conditioning (Bergstrom and Johnson 2014; LeDoux 2007; McDonald 2020) and appetitive memory (McDonald 2020). Excitatory neuron subtypes, such as SATB2 + and LAMP5 + are shared with the neocortex, though species-specific differences in their distribution are noted, with mice showing very little SATB2 + neurons in the LA, whereas the majority of these neurons in the BNC reside in this nucleus in humans (Yu et al. 2023). Humans also show a higher proportion of LAMP5 + neurons in the LA compared to the BA and BM, though in mice, these neurons are evenly distributed throughout the BNC nuclei (Yu et al. 2023). It is likely that this change between mice and humans occurred due to the increased size of the LA and neocortex in primates, with a corresponding increase in cell migration of pallial-derived excitatory neurons to this nucleus (Yu et al. 2023).

LA INs closely resemble those found in the neocortex and hippocampus, with distinct differences to those found in the BA nucleus (McDonald 2020). INs in this nucleus also exert a disinhibitory effect on INs in the CE via projection, which has been linked to the enhancement of the ‘fight or flight’ response (Šimić et al. 2021). In line with the auditory learning function of this nucleus, LA PV + INs uniquely receive feedforward inhibition from the auditory thalamus, a trait not seen in INs in other BNC nuclei (McDonald 2020). During fear conditioning in rodents, this excitatory drive onto LA INs is reduced and triggers the release of GABA, resulting in disinhibition of LA principal neurons and the acquisition of fear learning (McDonald 2020).

#### BA (basolateral nucleus of the BNC)

The basolateral nucleus (BA), consisting of the anterior (BLa), posterior (BLp), and ventral (BLv) subnuclei, located ventrally to the LA, shows the most similarities to the neocortex out of the BNC nuclei. It contains the largest neurons in the BNC, with the most morphological resemblance to those in the neocortex (McDonald 2020). In primates, it shows the highest correlation to the neocortex in terms of structure and function, especially in the realm of social function (Barton et al. 2003). For example, in macaques, single neurons in this nucleus showed preferential responses to faces and eye gaze, a trait also shared with the perirhinal and entorhinal cortices (Emery 2000). The BLa also plays a role in fear extinction, and auditory and visual processing in rats via projections received from the Te2 and auditory belt cortices (McDonald 2020). This commonality of visual processing in both rats and primates suggests an important role of the BLa subnucleus in assessing visual stimuli, which is of particular importance to social behaviours in primates, and has similarities to the visual processing function of layer IV of the neocortex.

The BA is characterised by its large number of cholinergic and dopaminergic innervations, and it is the main source of amygdala projections to the dorsal striatum and caudatus putamen (Martinez-Garcia et al. 2009; McDonald 1998; Olucha-Bordonau et al. 2015). These connections are remarkably symmetrical in both hemispheres, implicating this nucleus in the process of instrumental learning (Olucha-Bordonau et al. 2015). It is the primary convergence point for afferent projections from the prefrontal cortex, including the anterior insular, medial, and ventrolateral cortices, as well as being a target for projections from the entorhinal cortex, alongside the LaVM (Ghashghaei and Barbas 2002; Martinez-Garcia et al. 2009; McDonald 1998; Olucha-Bordonau et al. 2015; Pitkanen et al. 1997) (Fig. 3). These reciprocal connections between the PFC and BA are key to the extinction of fear memories, with lesions in the BA preventing fear extinction learning, whereas lesions in the mPFC prevent fear extinction memory recall (Olucha-Bordonau et al. 2015; Sierra-Mercado et al. 2011). Stimulation of the BLa induces an inhibitory response in the mPFC, which is also key to the control of the overall BNC in fear learning (Olucha-Bordonau et al. 2015). These prefrontal connections are remarkably conserved among mammals, including rats, cats and monkeys (Ghashghaei and Barbas 2002; Martinez-Garcia et al. 2009), highlighting the functional importance and age of the relationship between the PFC and amygdala. In primates specifically, the BA and BM also show strong, reciprocal connections to the posterior OFC (pOFC), which has implications for phobia and anxiety disorders (Ghashghaei and Barbas 2002; Kenwood et al. 2022). Labelled axon terminals originating from the OFC form a cluster in the BA, bordering the BM, and the medial edge of the LA, showing spatial segregation of projection neurons and axon terminals in particular nuclei (Ghashghaei and Barbas 2002). Within the BNC, the LA projects strong glutamatergic projections to the BA (Šimić et al. 2021) (Fig. 3). Similar to the connectivity and function of the LA, the BA also receives input from primary somatosensory areas, with specific neuron populations also responding to both appetitive and aversive learning and forming part of the associated engram (Janak and Tye 2015; Sah et al. 2003). However, unlike in the LaDl, efferent projections from these areas are unsegregated (Sah et al. 2003). Strong efferent projections emerge from this nucleus and the BM, and project to the neocortex, hippocampus, and ventral striatum, with the main amygdalostriatal pathway originating from the BA and BM nuclei (Martinez-Garcia et al. 2009; McDonald 1998; Olucha-Bordonau et al. 2015; Šimić et al. 2021) (Fig. 3). This pathway is especially important to reward learning and contextual fear conditioning, with lesions of the hippocampal connections preventing contextual fear learning (Martinez-Garcia et al. 2009; Olucha-Bordonau et al. 2015; Phillips and LeDoux 1994). The BA and BM are also important targets for the hypothalamus, whose projections terminate primarily in these nuclei (Ghashghaei and Barbas 2002; Pitkanen et al. 1997).

In contrast to the LA, BA subnuclei do not show strong reciprocated connections to each other, with the BLa being particularly selective in its projections to the other BA subnuclei (Pitkanen et al. 1997). This implicates that, in terms of function, individual BA subnuclei are more selective in the distribution and flow of sensory information throughout this nucleus, as well as to other areas external to the amygdala (Pitkanen et al. 1997). This ‘gatekeeping’ function and selectivity is reminiscent of the PFC’s wider inhibitory influence on the BNC, another similarity alongside the density of efferent projections received from this same area (Martinez-Garcia et al. 2009). The hub of intranuclei connections in the BA is the BLp, which projects strongly to both the BLa and BLv (though still less so compared to the intranuclei connections of the LA), and plays a key role in mediating activity between BA subnuclei, as well as conditioned fear response output from the entire BNC (Olucha-Bordonau et al. 2015; Pitkanen et al. 1997; Sah et al. 2003). It also shares neurochemical similarities to the adjacent anterior piriform cortex, which it is considered the deep pallial equivalent to, and shares an important function in the mediation of conditioned fear responses (Olucha-Bordonau et al. 2015). The BLv shows morphological similarities and a topographic position between the BLa subnucleus and the corticoamygdala transition zone (CxA), and can be considered intermediate between the two structures (Olucha-Bordonau et al. 2015). Similar to the relationship between the Blp and piriform cortex, the BLv could be considered the deep pallial equivalent of the CxA (Olucha-Bordonau et al. 2015). Information on its function relative to the rest of the BA remains scarce.

The principal neurons of the BA are the largest and closest to neocortical principal neurons out of the BNC, especially within the BLa (Fig. 4). Electron microscopy studies using CAMKII as a marker have shown that the synaptic organisation of inputs to principal neurons in the BLa are very similar to principal neurons in the neocortex (McDonald 2020). AChE staining of the BA reveals more intense staining compared to the LA, with the BLa and BLp showing the most intense reaction, and the BLv being comparatively faint, apart from a select population of rostrally-located neurons (Olucha-Bordonau et al. 2015). Parvalbumin (PV+) burst-firing INs in the BLa also show striking similarities to cortical PV + INs in firing and spike pattern, characterised by repetitive, high frequency discharge, followed by regular rhythmic firing patterns (McDonald 2020). Electron microscopy studies have shown this similarity extends to synaptic connectivity, with a similar network of PV + INs in both the BLa and neocortex showing electrical synaptic interconnectivity with each other (Hestrin and Galarreta 2005; McDonald 2020).

#### BM (basomedial nucleus of the BNC)

The basomedial nucleus, also known as the accessory basal nucleus, consisting of the anterior (BMa) and posterior (BMp) subnuclei, is the most ventral of the BNC nuclei (McDonald 2020). It primarily functions as a connector between the BNC and Ce and plays a strong influence on motivational behaviour via peptide secretions from its neurons, including corticotropin-releasing factor (CRF) and sex hormones such as oestrogen (Šimić et al. 2021). Alongside the BA and PFC, it also plays an important mediating role on the activity of the BNC, specifically in supressing activity relating to fear behaviour, as it is the main site of direct connections from the ventromedial PFC (vmPFC) (Šimić et al. 2021).

Unlike the other nuclei of the BNC, the BM remains largely isolated in rats, with its most prominent connections being between BM subnuclei (Pitkanen et al. 1997). As a result, information flow in the BM remains largely within the subnuclei and isolated from other nuclei, though it does receive strong glutamatergic projections from the LA nucleus (Pitkanen et al. 1997; Šimić et al. 2021) (Fig. 3).

Strong efferent projections emerge from this nucleus and the BA, and project to the neocortex, hippocampus, and ventral striatum (Ghashghaei and Barbas 2002; Šimić et al. 2021) (Fig. 3A). It also sends glutamatergic projections to the ventromedial hypothalamus via the stria terminalis, as well as the lateral hypothalamus (Martinez-Garcia et al. 2002, 2009) (Fig. 3). In rats, the BMa especially shares strong reciprocal projections to the olfactory cortex and cortical medial nuclei, particularly the anterior cortical nucleus (ACo), posterolateral (PLCo) and posteromedial cortical nuclei (PMCo), and the CxA (Olucha-Bordonau et al. 2015). Within the amygdala, the BMa and BMp both send afferents to the medial nucleus. The BM receives strong olfactory inputs from the piriform cortex and posterolateral and medial cortical amygdala (Ghashghaei and Barbas 2002; Martinez-Garcia et al. 2002). In macaques, there is also a heavy innervation of axonal terminals originating from visual temporal areas in this nucleus, which overlap with projection neurons connecting to the mPFC and OFC (Ghashghaei and Barbas 2002). Interestingly, despite the innervation and role of the BM in olfactory stimuli in rats, in terms of fear learning, the rat BMp shows increased activation (marked by c-fos expression) to visual stimuli (e.g. the sight of a predator) but not to associated olfactory stimuli (e.g. predator scent) (Olucha-Bordonau et al. 2015).

The principal neurons of the BM are the most divergent from the rest of the BNC (De Olmos et al. 1985; McDonald 2020) (Fig. 4). They are the smallest principal neurons in the BNC and show the most modifications to their features, with much fewer spines and sparser branching of the dendrites compared to the rest of the BNC (De Olmos et al. 1985; McDonald 2020). Within the BMa specifically, principal neurons show no dendritic orientation, another feature that diverges from the rest of the BNC, especially in other anterior subnuclei (De Olmos et al. 1985; McDonald 2020). However, these modified principal neurons, especially in the BMa, do show striking similarity to the equivalent neurons in the anterior cortex, and are especially difficult to distinguish between each other (De Olmos et al. 1985; McDonald 2020).

The BM contains fewer GABAergic INs compared to the other two BNC nuclei, though not as sparse as seen in the superficial layers of the neocortex (Martinez-Garcia et al. 2002). Neuron firing responses across principal neurons and INs are similar to those in the BLa and perirhinal and entorhinal cortices, marking close similarities between this nucleus and the neocortex, as well as a potential role in facial recognition (Emery 2000).

### Implications in mental health

The BNC and the cortex play a key role in emotional processing and regulation, and stress responses. The disruption of this relationship underpins multiple psychiatric disorders, especially in disordered emotional regulation involving fear, as well as substance abuse (Kim et al. 2011; LeDoux 2007; Levy and Schiller 2021; McDonald 2020; Šimić et al. 2021). There is evidence that this close relationship between the neocortex and BNC, especially in primates, while allowing for the development of crucial cognitive functions such as episodic memory and complex social abilities, also lead to the capacity for psychiatric disorders involving the emotions (Pine et al. 2021). This link is most implicated in anxiety disorders specifically (Kenwood et al. 2022), with BNC hyperactivity and mPFC hypoactivity being associated with fear generalisation, anxiety disorders, and learned helplessness (Kim et al. 2011; Levy and Schiller 2021; Šimić et al. 2021; Yu et al. 2023). Therefore, understanding this close relationship is crucial in bettering our understanding of the disorders that result when it is disrupted (Janak and Tye 2015; Mesulam 1998; Pabba 2013).

#### Anxiety, fear, and trauma

In generalised anxiety, BNC volume, activity, and connectivity to the dorsolateral PFC (dlPFC) is increased, with a corresponding decrease in overall PFC activity (Šimić et al. 2021). The resulting avoidant response (similar to the freezing response exhibited by rodents in fear conditioning models) is localised to the CE in humans, though there is evidence that this amygdala hyperreactivity is also driven by an imbalanced relationship between principal neurons and their INs in the LA (McDonald 2020; Šimić et al. 2021).

This same imbalanced relationship is also found in PTSD. The initial development of PTSD arises from the activation of the HPA axis and a cascading physiological response to acute stress (Šimić et al. 2021). When this stress response is prolonged, however, activity of INs in the LA is reduced, which results in an increased response in principal neurons, leading to an overall increase in anxiety (McDonald 2020; Šimić et al. 2021).

Imbalances in the LA also play a role in panic disorders. The volume of the right LA nucleus is significantly reduced to normal controls in people with panic disorder, and preclinical rodent models also show disruption in the functional connectivity between the LA and CE, including a reduction in LA IN density (McDonald 2020; Šimić et al. 2021).

#### Alcohol abuse

Alcohol disrupts the balance of excitatory and inhibitory transmission in the amygdala by increasing GABAergic transmission while decreasing glutamatergic transmission in the BNC and CE (Šimić et al. 2021). Prolonged alcohol exposure leads to a long-term increase in glutamatergic transmission and hyperactivity of pyramidal neurons in the BNC, leading to an increase in anxiety and consumption of alcohol (Šimić et al. 2021). It also disrupts the functional connectivity between the PFC and BNC, resulting in increased maladaptive social behaviours and a reduction in executive function (Šimić et al. 2021).

## Conclusion

This review aims to give an overview of the close relationship between the BNC and neocortex, including its role as a functional equivalent to the neocortex in animals that lack this structure. In doing so, we can see the close similarities between these two structures arising as a result of shared developmental origin, evolution, structure, and function in both learning and cognition. This link is crucial to further our understanding of both the amygdala and neocortex, particularly their close relationship in social behaviour and function, and their disruption in psychiatric disorders in humans. In bettering our understanding of these two areas as a functional and cohesive unit, it is hoped to improve targeting in treatments for psychiatric disorders involving both structures, and to better understand future directions of research in both.

## Data Availability

No datasets were generated or analysed during the current study.
